# 肠道组织宏基因组二代测序技术在单倍体造血干细胞移植后严重腹泻患者中的诊断价值

**DOI:** 10.3760/cma.j.cn121090-20241206-00540

**Published:** 2025-11

**Authors:** 巧贤 林, 晶晶 魏, 婷婷 连, 碧清 林, 金华 任, 晓云 郑, 雪琼 吴, 静 李, 涵 陈, 树俭 谢, 婷 杨

**Affiliations:** 1 福建医科大学附属第一医院滨海院区国家区域医疗中心血液科二科，福州 350212 The Second Department of Hematology, National Regional Medical Center, Binhai Campus of the First Affiliated Hospital, Fujian Medical University, Fuzhou 350212, China; 2 福建医科大学附属第一医院血液科二科，福州 350212 The Second Department of Hematology, the First Affiliated Hospital, Fujian Medical University, Fuzhou 350005, China; 3 福建医科大学附属第一医院血液科一科，福州 350005 The First Department of Hematology, the First Affiliated Hospital, Fujian Medical University, Fuzhou 350005, China; 4 福建医科大学附属第一医院滨海院区国家区域医疗中心内镜中心，福州 350212 Endoscopic Center, National Regional Medical Center, Binhai Campus of the First Affiliated Hospital, Fujian Medical University, Fuzhou 350212, China; 5 福建医科大学附属第一医院内镜中心，福州 350005 Endoscopic Center, the First Affiliated Hospital, Fujian Medical University, Fuzhou 350005, China; 6 福建医科大学精准医学研究院，福州 350001 Institute of Precision Medicine, Fujian Medical University, Fuzhou 350001, China

**Keywords:** 异基因造血干细胞移植, 严重腹泻, 分子诊断, 宏基因组二代测序技术, 感染性病原体, Allogeneic hematopoietic stem cell transplantation, Severe diarrhea, Molecular diagnosis, Metagenomics next generation sequencing Technology, Infectious Pathogens

## Abstract

**目的:**

分析肠道组织宏基因组二代测序技术（mNGS）在单倍体异基因造血干细胞移植（allo-HSCT）后并发严重腹泻或血便患者中的诊断价值。

**方法:**

纳入2023年6月19日至2024年8月30日在福建医科大学附属第一医院接受单倍体allo-HSCT后并发严重腹泻或血便的16例患者。所有患者均接受消化内镜检查，并通过mNGS检测肠道微生物基因。收集患者的内镜下表现、病理特征、病原微生物特征及临床预后数据，分析mNGS技术在感染性病原微生物诊断中的效能，以及基于检测结果调整治疗方案后腹泻症状的改善情况。

**结果:**

16例患者中，男12例，女4例，中位年龄32.5（3～60）岁。腹泻发生于造血干细胞回输后中位时间为3.93（1.63～10.40）个月。粪培养仅1例检出念珠菌，其余均为阴性；艰难梭菌抗原阳性1例，其余抗原及毒素检测均为阴性。内镜下肠道表现主要为充血、水肿、糜烂及出血，病理检查以黏膜局灶性炎性改变为主。mNGS检测显示87.5％（14/16）患者存在病原微生物感染，其中78.5％（11/14）为混合感染。常见病原体包括细菌（肺炎克雷伯菌、屎肠球菌、大肠埃希菌）、真菌（小孢根霉）及病毒（EB病毒、巨细胞病毒）。调整治疗方案后，87.5％的患者腹泻症状得到改善。

**结论:**

allo-HSCT患者因免疫抑制易并发感染性腹泻。在具备成熟内镜诊治技术的单位，通过mNGS技术对内镜活检组织进行分子诊断，有助于明确腹泻病因及感染病原微生物，指导针对性治疗，显著改善患者预后。

## Abstract (en)

**Objective:**

To evaluate the diagnostic value of intestinal tissue metagenomic next-generation sequencing（mNGS）in severe diarrhea following haploidentical allogeneic hematopoietic stem cell transplantation（allo-HSCT）.

**Methods:**

Sixteen patients who developed severe diarrhea or hematochezia after haploidentical allo-HSCT at the First Affiliated Hospital of Fujian Medical University（June 2023–August 2024）were enrolled. All underwent gastrointestinal endoscopy and mNGS for microbial detection. Clinical, endoscopic, pathological, and microbiological data were analyzed to evaluate the diagnostic value of mNGS and treatment outcomes following targeted therapy.

**Results:**

The study included 16 patients（12 males, 4 females; median age 32.5 years, range 3–60 years）. Diarrhea occurred a median of 3.93 months post-transplant（range 1.63–10.40 months）. Stool cultures were negative except for one case with Candida. One patient tested positive for Clostridium difficile antigen. Endoscopy revealed mucosal congestion, edema, erosion, and bleeding, with focal inflammation on pathology. mNGS detected pathogens in 87.5％（14/16）of cases, including mixed infections in 78.5％（11/14）. Common pathogens were Klebsiella pneumoniae, Enterococcus faecium, Escherichia coli, Rhizopus microsporus, EBV, and CMV. Targeted treatment adjustments led to symptom improvement in 87.5％ of patients.

**Conclusion:**

Allo-HSCT patients are prone to infectious diarrhea due to immunosuppression. Molecular analysis of endoscopic biopsy tissues using mNGS can accurately identify pathogens, guide targeted therapy, and improve clinical outcomes.

异基因造血干细胞移植（allo-HSCT）是治疗血液系统恶性疾病的重要手段。然而，移植后患者的免疫功能显著受抑，胃肠道并发症，尤其是严重腹泻成为影响患者生存质量和预后的关键问题[1]。研究表明，肠道菌群在维持肠道屏障功能、调节免疫反应以及抵御病原体感染方面发挥着重要作用[2]，而allo-HSCT后腹泻的发生常伴随肠道菌群的显著失衡，肠道菌群失调不仅导致肠道屏障功能减弱，还增加了感染性腹泻的发生风险[3]。传统的病原学检测方法（如粪培养）在识别复杂的混合感染或罕见病原体方面存在局限性[4]。近年来，宏基因组二代测序技术（Metagenomics next-generation sequencing, mNGS）的应用，为全面解析肠道菌群结构及病原谱提供了可能[5]–[6]，使得快速、精准地识别感染性病原体成为现实。

本研究旨在通过内镜下肠道组织标本的mNGS检测，探索其在单倍体allo-HSCT后严重腹泻患者中的诊断价值，并重点分析肠道菌群失衡与感染性病原体的关系。通过明确病因，为个体化治疗策略的优化提供科学依据，从而改善患者的临床预后。

## 病例与方法

1. 病例：本研究为回顾性分析，纳入了2023年6月19日至2024年8月30日期间在福建医科大学附属第一医院接受单倍体allo-HSCT后出现严重腹泻（即反复腹泻超过14 d且常规治疗无效）或排血便，需明确病因以控制症状的患者[7]。纳入标准：①年龄<60岁并接受过单倍体allo-HSCT；②合并严重腹泻：反复腹泻超过14 d且常规治疗无效）或排血便；③知情同意完善消化内镜采集肠黏膜组织样本并利用mNGS进行肠道病原学检测的患者。排除标准：①伴有精神疾病；②出现肝、肾、心等器官的功能衰竭；③伴有活动性的结核、肝炎等传染性疾病；④严重出凝血障碍合并全身多系统活动性出血；⑤妊娠或者哺乳期女性患者。本研究共纳入16例患者，所有患者均至少使用一种免疫抑制剂，包括糖皮质激素、环孢素A（CsA）、霉酚酸酯（MMF）及他克莫司。本研究已获得福建医科大学附属第一医院医学伦理委员会批准（批件号：[2015]084-2），所有患者均签署知情同意书。

2. 数据收集：系统收集患者的基本信息，包括年龄、性别、基础疾病、移植后腹泻发作时的临床状态，以及所用免疫抑制剂和化疗药物的种类及剂量。同时记录患者的中性粒细胞计数、血小板计数、凝血功能及腹泻发生时的其他临床指标。此外，还收集了腹泻期间的粪便培养结果、艰难梭菌毒素抗原检测结果以及通过消化内镜采集的肠黏膜组织mNGS检测结果，并记录各项检测结果对治疗方案的调整及腹泻的临床转归情况。

3. 造血干细胞移植方案：恶性血液病患者预处理使用FA5-BuCy方案，神经母细胞瘤（NB）患者采用RIC方案，其余患者则采用FCA方案，具体移植方案见文献[8]–[9]。所有患者均接受CsA、短程甲氨蝶呤（MTX）联合MMF预防移植物抗宿主病（GVHD）。细菌感染预防采用环丙沙星或左氧氟沙星，中性粒细胞减少期间或使用广谱抗生素前持续用药；抗真菌感染则根据指南采用氟康唑或其他抗霉菌药物（如泊沙康唑、伏立康唑），直至造血植入完成或患者因GVHD使用类固醇药物治疗时继续预防。巨细胞病毒（CMV）相关疾病的预防采用来特莫韦，移植后第28～100天内使用，并通过PCR监测CMV血清水平，阳性时启动更昔洛韦治疗。每周两次进行半乳甘露聚糖检测及CMV PCR分析，同时每周进行EB病毒（EBV）PCR监测，若发生EBV感染，则使用利妥昔单抗。总IgG水平低于4 g/L的患者，尤其是感染高风险者，可接受静脉注射免疫球蛋白（IVIG）替代治疗[10]–[13]。供者来源包括父母8例、同胞3例、子女5例。患者回输单个核细胞（MNC）中位数为9.19（2.87～19.06）×10^8^/kg，CD34^+^细胞中位数为5.12（2.00～15.03）×10^6^/kg。中性粒细胞和血小板的中位植入时间分别为15（11～21）d和13.5（11～50）d。

4. 消化内镜采集肠黏膜组织样本：所有患者在检查前均签署了知情同意书，以确保充分了解检查的必要性及可能风险。检查使用OLYMPUS CLV-290SL内镜系统，由经验丰富且具有中级及以上职称的消化内科医师完成操作。

5. mNGS检测：肠黏膜组织样本在无菌条件下保存于−20 °C，并送至予果生物科技（北京）有限公司进行mNGS检测。检测范围覆盖3万余种已知微生物基因组序列，包括细菌17 748种、病毒11 058种、真菌1 134种及寄生虫308种。DNA提取和纯化参照QIAamp DNA Micro Kit操作说明，通过Qubit 3.0荧光计和琼脂糖凝胶电泳检测DNA浓度和质量。合格DNA文库采用Illumina Nextseq 550测序平台（SE75bp策略）进行测序，平均数据量不低于20 M。测序数据经过滤处理后，通过SNAP软件去除人源序列，并利用Burrow-Wheeler Alignment软件将剩余数据与NCBI微生物基因组数据库比对。阳性判定标准包括：①细菌、真菌及寄生虫：测序覆盖率排名前10且在阴性对照中未检出，或样品/阴性对照RPM比值大于10；②病毒、结核杆菌及隐球菌：至少检出1条特异性序列且阴性对照未检出，或样品/阴性对照RPM比值大于5。

6. 耐药性检测：耐药基因检测与宏基因组测序同步进行，结果与CARD数据库进行比对以确认耐药基因片段。

7. 粪培养及鉴定：新鲜粪便样本接种后利用飞行质谱技术进行菌种鉴定。

## 结果

1. 患者基本情况：本研究共纳入16例患者，其中男12例（75％），女4例（25％），中位年龄为32.5（3～60）岁。患者在allo-HSCT后出现严重腹泻或血便，发病时间距造血干细胞回输的中位时间为3.93（1.63～10.40）个月。原发疾病包括急性髓系白血病（AML）8例（50％）、骨髓增生异常综合征（MDS）2例（12.5％）、急性淋巴细胞白血病（ALL）1例（6.25％）、重型再生障碍性贫血（SAA）1例（6.25％）、淋巴瘤3例（18.75％）及NB 1例（6.25％）。其中，1例患者合并噬血细胞综合征并正处于化疗过程中，其余患者均未接受化疗。消化内镜检查时，患者的中位中性粒细胞计数为1.60（0.06～5.66）×10^9^/L，中位血小板计数为56.5（14～173）×10^9^/L，中位活化部分凝血活酶时间（APTT）为30.95（12.6～77.5）s，中位凝血酶原时间（PT）为11.6（10.0～25.5）s。尽管存在血小板减少和凝血功能异常，大多数患者仍完成了内镜检查。病例资料详见表1。

**表1:** 16例单倍体造血干细胞移植后合并严重腹泻患者的特征

序号	年龄（岁）	性别	诊断	中性粒细胞计数（×10^9^/L）	严重腹泻/血便	GVHD	基于mNGS结果	病理	治疗方案调整	改善时间（d）
1	50	男	sAML	1.06	严重腹泻	否	大肠埃希菌	黏膜局灶炎	停环孢素，加强抗细菌	2
2	60	女	MDS	1.51	血便	是	阴性	大部分隐窝上皮坏死消失	加巴利昔单抗	2
3	27	男	SAA	5.66	严重腹泻	否	结核杆菌	黏膜活动性炎	抗结核	7
4	3	女	AML	0.86	血便	否	微小根毛霉、巨细胞病毒、EB病毒	黏膜慢性炎	停免疫制剂，加泊沙康唑及更昔洛韦	5
5	42	男	MDS	2.15	严重腹泻	否	屎肠球菌、肺炎克雷伯菌、微小根毛霉、小孢根霉、巨细胞病毒	符合巨细胞病毒感染，黏膜局灶活动性炎	加更昔洛韦	3
6	45	男	PTCL	1.69	血便	是	肺炎克雷伯等细菌、微小根毛霉、人类疱疹病毒1	黏膜慢性炎	抗细菌	4
7	15	男	AML	4.60	严重腹泻	是	小孢根霉、EB病毒	黏膜活动性炎伴糜烂、隐窝炎隐窝脓肿。符合GVHD	加两性霉素B、利妥昔单抗	3
8	40	女	AML	1.10	严重腹泻	是	肺炎克雷伯、小孢根霉	黏膜慢性炎	加两性霉素B	3
9	37	男	AML	2.13	严重腹泻	否	肺炎克雷伯等细菌、总状毛霉、小孢根霉、EB病毒	糜烂渗出肉芽增生	加两性霉素B、丙种球蛋白、利妥昔单抗	10
10	17	男	NK/TCL	0.06	血便	是	屎肠球菌、人哺乳动物腺病毒C型、EB病毒	糜烂肉芽肿形成少量细胞非典型未进一步检查	加丙种球蛋白、加强抗细菌	无
11	7	男	NB	2.29	严重腹泻	否	总状毛霉、小孢根霉、EB病毒	未检测	加两性霉素B，停更昔洛韦	7
12	32	男	AML	1.75	严重腹泻	是	肺炎克雷伯菌、小孢根霉	黏膜局灶性炎	加两性霉素B、加抗细菌	7
13	18	男	AML	0.16	严重腹泻	是	大肠埃希菌、腺病毒	未检测	加抗细菌	5
14	33	男	NHL	1.04	血便	是	鲍曼不动杆菌	黏膜慢性炎伴糜烂	加抗细菌及巴利昔单抗	20
15	39	男	AML	0.54	严重腹泻	是	屎肠球菌等细菌	未检测	加抗细菌及丙种球蛋白	无
16	14	女	ALL	2.03	严重腹泻	是	阴性	黏膜局灶活动性炎	抗细菌	3

**注** sAML：继发性急性髓系白血病；MDS：骨髓增生异常综合征；SAA：重型再生障碍性贫血；AML：急性髓系白血病；PTCL：外周T细胞淋巴瘤；NK/TCL：NK/T细胞淋巴瘤；NHL：非霍奇金淋巴瘤；ALL：急性淋巴细胞白血病；GVHD：移植物抗宿主病；mNGS：宏基因组二代测序

2. 粪便检查及肠组织经mNGS技术检测结果：

（1）粪培养：仅1例患者检出念珠菌，其余15例均为阴性。

（2）粪艰难梭菌抗原和毒素：艰难梭菌抗原及毒素检测中，1例患者艰难梭菌抗原阳性，其余均为阴性。

（3）mNGS结果：所有16例患者的肠黏膜组织标本均在24～48 h内获得检测结果，共检出45株病原微生物，包括细菌20株、真菌11株和病毒14株。其中，细菌以肺炎克雷伯菌（6株，中位序列数19 079.5）、屎肠球菌（3株，中位序列数51 378）及大肠埃希菌（3株，中位序列数63）为主；真菌以小孢根霉（6株，中位序列数348.5）、微小根毛霉（3株，中位序列数307）及总状毛霉（2株，中位序列数3 061）为主；病毒主要为EBV（中位序列数19.5）和CMV（中位序列数7 938.5）。此外，还检测到1株结核杆菌。在16例送检样本中，14例（87.5％）检出潜在致病微生物，其中2例（12.5％）检出1株，5例（31.2％）检出2株，2例（12.5％）检出3株，5例（31.2％）检出4株及以上。

3. 内镜结果及病理表现：16例患者中有10例发现不同程度的黏膜糜烂与溃疡，其中部分患者表现为弥漫性溃疡并伴随出血；2例表现为红肿及红斑；其余4例未发现明显异常。病理检查方面，13例患者成功完成组织病理检查，结果显示2例存在隐窝减少或隐窝炎症及脓肿，1例病理特征符合CMV肠炎，其余患者主要表现为黏膜炎。

4. 药敏分析结果：6株肺炎克雷伯菌中2株对氨基糖苷类、四环素类、头孢菌素类和磺胺类抗生素耐药，1株对青霉烯类、氟喹诺酮类、林可胺类、氯霉素、磷霉素耐药。3株屎肠球菌均对氨基糖苷类耐药，2株对四环素类和林可胺类抗生素耐药，1株对头孢菌素、磺胺、碳青霉烯、氟喹诺酮及大环内酯类抗生素耐药。3株大肠埃希菌中1株对四环素类及氨基糖苷类抗生素耐药。

5. 治疗与转归：患者接受了经验性止泻、抗排异及头孢类抗感染治疗，并根据mNGS结果进行针对性的抗感染治疗，包括抗结核、抗EBV、抗CMV以及抗细菌/真菌治疗。最终，14例患者的腹泻症状均显著改善，排便次数减少一半以上，大便成型且无血便，中位改善时间为4（2～20）d。但仍有2例患者无明显改善，其中1例疑似肿瘤复发，另1例考虑合并肝脏等其他GVHD，且其凝血功能恶化进展。

## 讨论

allo-HSCT是治疗恶性血液系统疾病的重要手段，尽管其疗效显著，但移植后胃肠道并发症，尤其是腹泻，仍是临床管理中的一大难题。移植后腹泻的成因复杂，涉及GVHD、感染、药物毒性等多种因素，且鉴别诊断困难[14]。本研究结合内镜观察、组织病理学分析及mNGS技术，对严重腹泻或血便患者进行诊治后发现，大部分患者存在潜在致病菌感染。根据mNGS结果调整治疗方案后，约87％的患者症状显著改善，显示出mNGS在难治性腹泻及血便病因诊断中的重要价值。

既往文献报道，移植后胃肠道GVHD是移植后腹泻的重要病因之一[15]。值得注意的是，GVHD与感染并非完全对立关系，其间复杂的相互作用可能与病原学检出敏感性有关。在GVHD、免疫抑制治疗、长期住院、广谱抗生素应用及化疗毒性等多重因素作用下，胃肠道屏障功能受损，增加了感染性腹泻的风险[16]–[17]。本研究显示，病原体种类包括细菌、病毒及真菌，其中以CMV感染最为突出。然而，便血和外周血CMV-DNA对CMV肠炎的诊断意义有限[18]，而mNGS技术通过即时反馈显著缩短了诊断时间，并优化了治疗策略。

本研究进一步证实了感染性腹泻对移植后患者早期生存率的负面影响。感染性腹泻不仅增加非复发死亡率[17],[19]，还与血流感染风险密切相关[20]。传统粪培养方法难以明确致病菌，而基于mNGS的组织标本检测显示出更高的病原体检出率和精准性[6]。本研究中，主要检出的细菌包括肺炎克雷伯菌、屎肠球菌及大肠埃希杆菌，病毒以EBV和CMV为主，真菌则以小孢根霉及总状毛霉为主。尤其是在部分病例中，组织病理学检查结果回报时间较长，而mNGS结果在24～48 h内即可获得，显著提高了诊断效率。

此外，本研究提示移植后患者肠道真菌感染需要特别关注，尤其是根霉等非曲霉属丝状真菌（Non-Aspergillus filamentous fungi，NAMI）感染。NAMI感染具有高致死率，其危害远高于曲霉菌感染[21]–[23]。现有研究样本量虽较小，但NAMI的高耐药率和高病死率表明，早期诊断和治疗对于管理此类感染至关重要。相较于传统方法，mNGS技术展现出在快速识别罕见病原体方面的显著优势，为精准治疗提供了重要参考。

本研究的局限性主要包括以下几点：首先，样本量较小，但由于针对肠道组织的mNGS检测文献较少，本研究仍具有重要参考价值；其次，未设立对照组，无法完全客观评估检测技术的准确性；此外，并非所有患者均接受病理组织检查，但这对治疗和结局的影响有限；最后，部分患者未进行结肠镜检查，在严重腹泻及血便患者中，结肠镜检查显著提升了诊断和治疗的效果。

综上所述，allo-HSCT后患者因免疫功能受抑，感染性腹泻是难治性腹泻的主要病因，且存在多种潜在病原体感染的风险。在内镜诊治技术成熟的单位，结合基于mNGS的组织分子诊断技术，有助于明确感染相关病原微生物，及时调整抗菌治疗方案，从而改善患者预后。这一研究为移植后腹泻的精准诊治提供了重要参考，并为未来更大规模的研究奠定了基础。
